# Workplace-Related Interpersonal Group Psychotherapy to Improve Life at Work in Individuals With Major Depressive Disorders: A Randomized Interventional Pilot Study

**DOI:** 10.3389/fpsyt.2020.00168

**Published:** 2020-03-17

**Authors:** Daryl Wayne Niedermoser, Nadeem Kalak, Anna Kiyhankhadiv, Serge Brand, Corinna Walter, Nina Schweinfurth, Undine E. Lang

**Affiliations:** ^1^University Psychiatric Clinics (UPK), University of Basel, Basel, Switzerland; ^2^Departement of Business, Kalaidos University of Applied Sciences, Zurich, Switzerland; ^3^Substance Abuse Prevention Research Center, Kermanshah University of Medical Sciences, Kermanshah, Iran; ^4^Department of Sport, Exercise and Health, University of Basel, Basel, Switzerland; ^5^School of Medicine, Tehran University of Medical Sciences, Tehran, Iran; ^6^Sleep Disorders Research Center, Kermanshah University of Medical Sciences, Kermanshah, Iran

**Keywords:** workplace-related stress, interpersonal psychotherapy, depression, ability to work, self-efficacy to returning to work, sleep

## Abstract

**Objectives:**

Individuals suffering from major depressive disorder (MDD) often report workplace-related stress as the major cause of their disorder. Accordingly, workplace-related stress was established as a fifth psychosocial problem area of Interpersonal Psychotherapy (workplace-related Interpersonal Psychotherapy, W-IPT). The aim of the study was to investigate the influence of W-IPT on depressive symptoms and on workplace-related issues in individuals with MDD compared to a treatment-as-usual (TAU) condition.

**Methods:**

A total of 27 individuals with MDD (mean age = 43 years, 48% males) were randomly assigned either to eight weekly group sessions of W-IPT or to the TAU condition. At baseline, 8 weeks later at the end of the intervention, and 20 weeks later at follow-up, the Hamilton Rating Scale for Depression was conducted. In addition, the participants completed the Beck Depression Inventory, the Work Ability Index (WAI), the Return to Work Attitude (RTW-SE), and the Insomnia Severity Index (ISI).

**Results:**

Symptoms of depression in experts' ratings as well as in self-rated ratings decreased over time, but more so in the W-IPT condition compared to the TAU condition [experts rating: large effect size (*d* = 1.25) and self-assessment: large effect sizes (*d* = 0.94)]. The subjective ability to work (WAI) [medium effect size (*d* = 0.68)], self-efficacy to returning to work RTW-SE [medium effect size (*d* = 0.57)], and subjective symptoms of insomnia (ISI) [large effect size (*d* = 1.15)] increased over time, but again more so in the W-IPT condition compared to the TAU condition. The effects of the intervention remained stable from the end of the intervention to follow-up.

**Conclusions:**

The pattern of results of this pilot study suggests that a newly established fifth IPT focus on workplace-related stress appeared to be particularly efficient in individuals with MDD due to work-related stress in reducing depressive symptoms and reducing sleep complaints as well as in improving occupational outcomes.

## Introduction

Major depressive disorder (MDD) is a common and disabling psychiatric disorder associated with an increase in disability and mortality (1). Based on the data using the disability-adjusted-life-years to assess “the sum years lost due to premature mortality and years lived with disability adjusted for severity,” Murray and Lopez (2) estimated that MDD will be the third leading cause of burden worldwide by 2020. By contrast, Jorm et al. (3) claimed that the epidemiologic prevalence rates of MDD did not increase within the 30 years, while the social awareness of depression did.

Standard treatments of moderate and severe MDD consist mainly on the administration of antidepressants, *i*.*e*., serotonin-reuptake inhibitors. More recent reviews and meta-analyses questioned the efficacy of antidepressants and the unpleasant side effects might be a reason why people with MDD often quit antidepressant medication (4–8). Other evidence-based treatment options are neuromodulation (9–11), cognitive–behavioral interventions (12, 13), or regular physical activity (14–17).

Treatment guidelines (AWMF, NICE) recommend antidepressants and/or psychotherapy to treat moderate and severe MDD. Individuals with major depressive disorders often report stressful issues related to the workplace. Job strain, low job control, low social support, high psychological demands, effort–reward imbalance, and high job insecurity were confirmed as predictors particularly for depression (18–23). Several studies (24–27) show that, compared to healthy individuals, individuals with MDD are at an increased risk to lose their current job position (24, 25, 27), to have more difficulties to go back to their workplace (24, 25, 27), to find a new job, once they have been dismissed, and to keep their job position, when they return to their job after a period of illness-related unemployment. Furthermore, data on health costs show that, compared to an accident-related absence from work, depression-related absence from work cause higher economic burden for the individual (25, 27), the employer (27–29), and the public (24, 26, 27, 30). In addition, losing a job position or experiencing difficulties to go back to work turned out to be a risk factor for further relapses of MDD (24).

In this context, specific interventions to reduce workplace-related stress in individuals with MDD might both reduce the economic costs for the public and the burden for the individual (24, 26, 27, 29, 30).

Here we present the influence of the newly developed workplace-related Interpersonal Psychotherapy (W-IPT) which is a specific intervention to influence the workplace-related dimensions of symptoms of individuals with MDD. The original IPT concept was developed as a brief psychotherapeutic treatment aiming at the symptoms of an acute depressive disorder and current interpersonal problems and its effectiveness for the treatment of MDD has been widely demonstrated in numerous controlled trials [cf. meta-analysis from Cuijpers et al. (31)].

The W-IPT focuses on issues occurring in the context of the work role (32) such as social and interpersonal problems at work, mobbing, interpersonal conflicts, role transition, role confusion, burnout, boreout, job strain, low social support, effort–reward imbalance, job demand–control imbalance, and work–life imbalance.

The following three hypotheses and one research question were formulated and each of these is considered in turn. First, following Knekt et al. (33), Hange et al. (34), and Hallgren et al. (35), we expect that, compared to the TAU condition, the workplace-related dimensions of (a) the ability to work and (b) the self-efficacy in returning to work increased over time. The second hypothesis was in the context of empirical evidence (12, 31, 36, 37). We expect that the W-IPT treatment has positive effects on depressive symptoms when compared to the TAU condition. The symptoms of depression will improve in the experts' rating and in the self-rating. Following the second hypothesis and combining it with those of Santor and Kusumakar (38) and Lemmens et al. (37), we formulated the following exploratory research question. If there is a benefit in the W-IPT group, is the effect stable over a 3-month period measured at follow-up? Third, following Göder et al. (39) and Dombrovski et al. (40), we expected that, compared to the TAU condition, the W-IPT treatment will decrease the symptoms of insomnia.

## Method

### Procedure

We conducted a monocentric, randomized, controlled trial comparing W-IPT *versus* TAU condition in a group format in outpatients with major depression related to workplace-related issues between April 2018 and September 2019. The regional ethics commission (Ethikkommission Nordwest- und Zentralschweiz) approved the study (application number 2017-01-489). The eligible patients were fully informed about the study and signed the written informed consent at the first meeting. Moreover, all procedures were in line with the ethical standards of the Declaration of Helsinki (41) and its later amendments and with the ethical code of conduct of the American Psychological Association.

**Figure 1:**
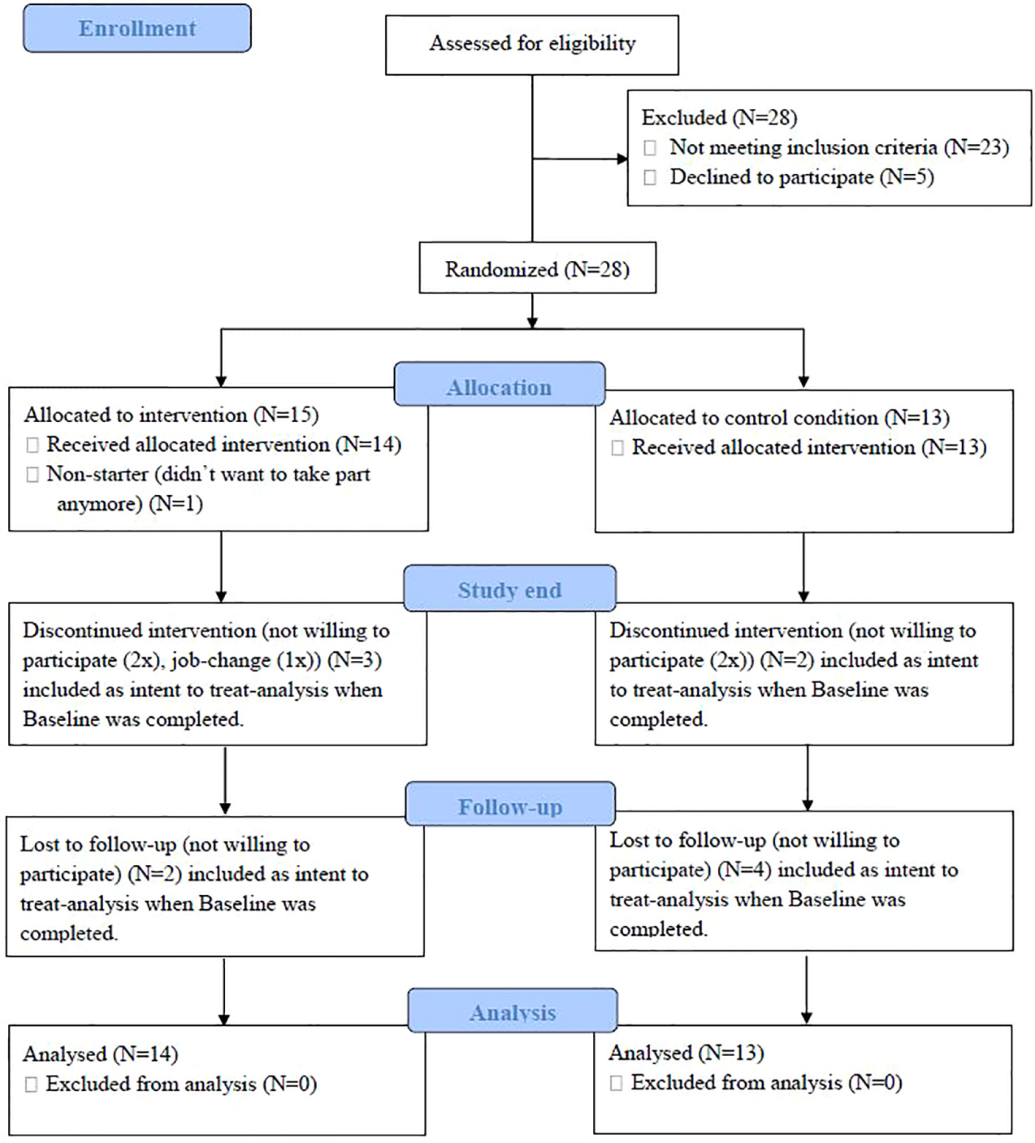
CONSORT flow diagram.

There was a total of one individual screening before the baseline and three data collections. The Hamilton Experts' Rating Scale for Depression (HRSD-24) was conducted in the screening part before the baseline as an information guide whether to include or exclude participants and to train (and explain) the questions. At baseline, the HRSD-24 was conducted and all questionnaires were filled out by the participants. The same procedure was applied at the last meeting (8 weeks after the start of the intervention) and at 12 weeks after the end of the treatment (follow-up at 20 weeks after randomization) (see **Figure 1** and **Table 1**).

**Table 1:** Overview of use and use of the questionnaires.

Questionnaires	Prestart	Start	Study-end	Follow-up
SCID-I	X			
Informed Consent	X			
Sociodemographics	X			
Hamilton 24	X	X	X	X
Becks Depression Inventory		X	X	X
Work Ability Index		X	X	X
Return to Work		X	X	X
Insomnia Severity Index		X	X	X

Prestart = initial consultation, SCID-I, Structured Clinical Interview DSM-IV.

### Sample

In-patients and out-patients were recruited through either a media announcement or a licensed practitioner. A total of 56 interested participants were clarified after a quick telephone/e-mail screening. They were invited to an extended interview [interview, informed consent, Structured Clinical Interview for Disorders on the axis I (SCID-I), and HRSD-24]. A total of 28 (50.0%) of the 56 patients fulfilled the inclusion criteria. The inclusion criteria were (1) being between 18 and 65 years of age, (2) major depressive disorder (SCID-I), (3) issues at or with the workplace (*e*.*g*., mobbing, effort–reward imbalance, and few social support), (4) fluent in German, (5) signed written informed consent, (6) complying with the study conditions, and (7) no or stable psychopharmacological and psychotherapeutic treatments 4 weeks before the study and throughout the study. The exclusion criteria were (1) HRSD-24 < 18), (2) acute suicidality, (3) psychotic symptoms or bipolar disorder, (4) primary diagnosis of another mental illness, (5) substance use disorder, (6) personality disorder, and (7) applied (or already accepted) disability pension.

Of the 28 eligible participants, there was one non-starter and 27 started (mean age = 43 years, SD = 10.2, 48% males) participating in the present study. There were 14 participants assigned to the W-IPT and 13 to the TAU condition (see **Figure 1**, CONSORT flow chart).

### Randomization

A psychologist not otherwise involved in the study performed the randomization. A total of 15 blue and 15 red chips were put in an opaque ballot box and stirred. The psychologist draws a chip and assigned the participant to the study condition. Then, the chip was put aside. To make sure that the blinding procedure is still clear, the participants were informed not to tell the psychologist anything about their treatment. After having taken part in the TAU condition, the participants were given the option to participate in a W-IPT group. The two groups did not differ as regards to age range, gender, civil status, employment, highest educational qualifications, antidepressants use, or in- and outpatient (see **Table 2**).

**Table 2:** Sociodemographic and illness-related information, separately for participants in W-IPT and TAU condition.

	Groups		Statistics
	W-IPT	TAU	
N	14	13	
Age range in years	24–55	28–55	
Age in years M (SD)	40.86 (11.29)	45.77 (8.438)	*t*(25) = -1.279, *p* = .213
Gender (female/male)	7/7	7/6	*X^2^*(*N*=27 *df* = 1) = 0.04, *p* = .84
Civil status (single/married)	10/4	9/4	*X^2^*(*N*=27, *df* = 1) = 0.16, *p* = .90
Current job position (yes/no)	11/3	9/4	*X^2^*(*N*=27, *df* = 1) = 0.31, *p* = .58
Educational level (high school/diploma/university degree)	1/6/7	1/5/7	*X^2^*(*N*=27, *df* = 2) = 0.05, *p* = .97
Antidepressants (yes/no)	10/4	7/6	*X^2^*(*N*=27, *df* = 1) = 1.82, *p* = .18
Participants (In-/Outpatient)	7/7	4/9	*X^2^*(*N*=27, *df* = 1) = 0.93, *p* = .34

### Measurements

To test our hypothesis, we used internationally recognized and well-established questionnaires in German. For several hypotheses, we used a few less-known questionnaires. These have been translated and translated back to ensure that the content is queried correctly. Mental disorders were assessed by means of the SCID-I based on DSM-IV (42, 43).

### Depression Questionnaires

#### Experts’ Rating

The Hamilton Rating Scale for Depression (44) consisted of 24 items, asking on low mood, suicidality, irritability, tension, loss of appetite, loss of interests, and other somatic symptoms. Answers were given on rating scales differing from three-, four-, or five-point ratings. Higher sum scores reflect more marked depressive symptoms. Additionally, the scores were categorized as follows: 0–7, no depressive symptom/remission; 8–17, mild; 18–24, moderate; 25 and more, severe depressive disorder (Cronbach's alpha = 0.71).

#### Self-Rating

The Beck's Depression Inventory (BDI-II) examines the depressive symptomatology from a self-perspective (45). The BDI-II is a widely established self-assessment tool with 21 items. It captures the affective, cognitive, and behavioral dimensions as well as the somatic symptoms of depression. The test quality criteria, such as internal consistency, validity, and test–retest reliability, are most satisfactory in both clinical and non-clinical subjects (46) (Cronbach's alpha = 0.89).

#### Workplace-Related Questionnaires

The Work Ability Index (47) is a measurement tool that uses a questionnaire to calculate an index value for assessing the ability to work. This index value indicates the extent to which an employee is able to carry out his/her work on the basis of his/her personal circumstances and existing working conditions (Cronbach's alpha= 0.70).

Return to Work Self-Efficacy (RTW-SE) is the belief in one's own ability to meet the demands required to return to work. The “return to work attitude” questionnaire (48) contains 11 questions on self-efficacy in managing demands at work (Cronbach's alpha = 0.66).

### Subjective Sleep Quality

#### Sleep Complaints Measured With the Insomnia Severity Index

The questionnaire is in part in line with the DSM-IV insomnia criteria and measures current perception (within the last 2 weeks) in terms of symptom severity, stress, and impairment. These include severity of onset and persistence of sleep (mid-morning and early morning wake disorder), satisfaction with the current sleep pattern, daily life impairment, occurrence of impairments due to sleep problem, and concern about sleep disorders [see Morin et al. (49) and Gerber et al. (50)] (Cronbach's alpha = 0.77).

### Intervention

#### Work-Related Interpersonal Psychotherapy

The W-IPT condition followed a guideline (51, manuscript in preparation). W-IPT focuses on the work context by adding specific elements to the regular IPT strategies: identifying work-related stress factors using an individual stress and resource profile, creating a balance between performance values and interpersonal values, teaching mindfulness and coping strategies for work-related stress (including social support), and practicing social communication skills at work to cope with conflict and role changes. By applying those strategies, we address social and interpersonal problems at work, such as mobbing, interpersonal conflicts, role transition, role confusion, burnout, boreout, job strain, low social support, and work–life imbalance (for more details, see **Table 3**). The intervention includes one weekly group session of 90 min for six to eighr participants over 8 weeks in addition to the TAU condition. Trained psychotherapists, certified in IPT treatment, conducted the group sessions.

**Table 3:** Content of the sessions on workplace-related interpersonal psychotherapy.

Sessions	Content of sessions
One	Introducing members, identify work-related stress and dysbalances in the context of depression, theory and information, mindfulness exercise, providing handouts and homework
Two	Reflecting and discussing the homework, thoughts and feelings, introduction to self-care and mindfulness, role play exercise, and quick meditation, providing handouts and homework
Three	Reviewing homework, interpersonal stress and conflicts, introducing the Kiesler circle model, role play exercise, do not fall victim, mindfulness meditation, providing handouts and homework
Four	Staying in the present, mindfulness exercise of the body scan, communication at the workplace, how to solve conflicts at the workplace (using the Kiesler model), discussing the homework, providing handouts and homework
Five	Reflecting and discussing the homework, define and identify the living values, handout and discussion of what's important to me, building a supportive network, work–life balance, self-compassion exercise, providing handouts and homework
Six	Mindfulness exercise, reviewing the homework, role play, to ask for help, providing handouts and homework
Seven	Mindfulness exercise, implementation of the values at the workplace, change and acceptance, providing handouts and homework
Eight	Reviewing and discussion of the homework, balance between acceptance and change, applying what was learned, conducting a survey of the course, conclusion and finishing the course along with last contemplation

### TAU Condition

The participants randomized to the TAU condition received no further intervention during the study period and were encouraged to continue with attending appointments with their clinicians. In order to compensate for non-participation in the W-IPT condition, the patients in the TAU condition were offered to participate in the W-IPT program after their follow-up evaluation.

### Statistical Analysis

Following Julious (52), a caseload of 12 individuals per group in pilot studies is reasonable to start with. A series of t-tests and Pearson's correlations was performed to compare socio-demographic, workplace-related, depression-related, and sleep complaints information between the two groups.

To assess changes over time and between and within the two groups, a series of ANOVAs for repeated measures was performed with the factors time (start, study end, and follow-up), group (intervention and TAU), and the time by group interaction and with symptoms of depression (experts' ratings and self-ratings), work-related information, and subjective sleep complaints as dependent variables. Due to the deviation of sphericity, the ANOVAs were performed using Greenhouse–Geisser corrected degrees of freedom, although the original degrees of freedom are reported with the relevant Greenhouse–Geisser epsilon value (*ϵ*). All computations were performed with the intention-to-treat analysis, with the last observation carried forward method. For ANOVAs, the effect sizes were reported as partial eta squared (η_p_^2^), with 0.01 < η_p_^2^ < 0.059 indicating small (S), 0.06 < η_p_^2^ < 0.139 indicating medium (M), and η_p_^2^ > 0.14 indicating large (L) effect sizes. In addition, we followed Becker (53) and reported effect sizes for t-tests within and between the groups for all time points. To classify the effect sizes, we followed Cohen (54): effect sizes can be evaluated as trivial (T; *d* = 0–0.19), small (S; *d* = 0.20–0.49), medium (M; *d* = 0.50–0.79), or large (L; *d* = 0.80 and greater). The level of significance was set at alpha *p* ≤.05. All statistical calculations were performed with SPSS^®^ 25.0 (IBM Corporation, Armonk, NY, USA) for Windows^®^.

## Results

**Figure 2:**
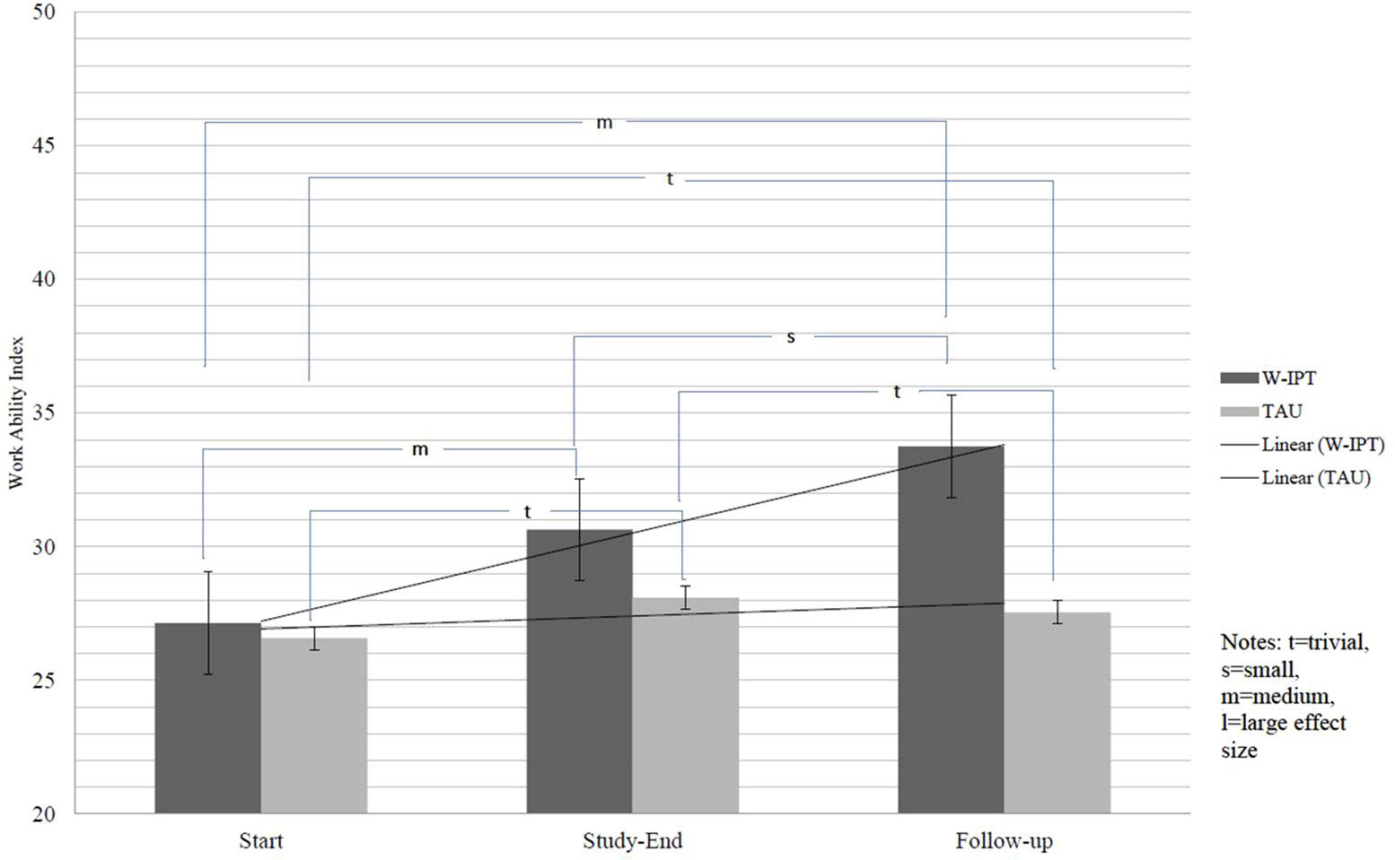
Work Ability Index (WAI).

All descriptive, inferential, and statistical indices are reported in **Tables 4**–**6**, and **Figure 2**. **Table 4** shows the descriptive statistics, **Table 5** shows the inference statistics, **Table 6** shows the effect size comparisons, and **Figure 2** shows the work ability index.

**Table 4:** Descriptive overview of the descriptive statistics, separately for assessment time (start, study-end, and follow-up) and group (W-IPT vs. TAU condition).

	Assessment times					
	Baseline		Study-end (+ 8 weeks)		Follow-up (Total of 20 weeks)	
	W-IPT (*N*=14)	TAU (*N*=13)	W-IPT	TAU	W-IPT	TAU
	M (SD)	M (SD)	M (SD)	M (SD)	M (SD)	M (SD)
Hamilton 24	25.5 (4.88)	27.46 (12.18)	16.36 (9.16)	23.85 (15.88)	16.29 (10.62)	22.39 (14.38)
Becks Depression Inventory	24.07 (8.11)	28.23 (12.23)	16.14 (8.74)	21.92 (13.81)	15.71 (10.74)	20.85 (12.50)
Work Ability Index	27.14 (10.48)	26.58 (7.80)	30.64 (10.12)	28.08 (10.22)	33.75 (9.58)	27.54 (9.50)
Return to Work	28.21 (5.22)	27.62 (6.23)	31 (4.56)	27 (6.30)	31.79 (5.45)	28.15 (6.89)
Insomnia Severity Index	14 (5.19)	14.67 (5.71)	8.64 (4.11)	13.31 (7.25)	7.79 (5.37)	12.23 (7.11)

**Table 5:** Inferential statistics of depression, work, and sleep, with the factors Time (start, study-end & follow-up), Group (intervention vs. TAU), and the Time × Group interaction.

	Factors						
	Time		Group		Time × Group		Greenhouse–Geisser
	*F*	η_p_^2^	*F*	η_p_^2^	*F*	η_p_^2^	Epsilon
Hamilton 24	12.048***	0.325 [L]	1.610	0.060 [M]	1.618	0.061 [M]	.995
Becks Depression Inventory	15.083***	0.376 [L]	1.674	0.063 [M]	0.133	0.005 [S]	.854
Work Ability Index	3.904*	0.135 [M]	4.981*	0.166 [L]	2.159	0.079 [M]	.976
Return to Work	3.120	0.111 [M]	1.843	0.069 [M]	2.571	0.093 [M]	.955
Insomnia Severity Index	12.616***	0.335 [L]	2.676	0.097 [M]	3.032	0.108 [M]	.706

Degrees of freedom—Time: (2, 25), Group: (1, 25), Time × Group (1, 25). S, small effect size; M, medium effect size; L, large effect size. S > 0.01, M > 0.06, L < 0.14. *p < 0.05; ***p < 0.001.

**Table 6:** Effect sizes for mean comparisons form pre- to post-assessment within the groups (intervention group and TAU group).

Group	Start to study-end		Start to follow-up		Study-end to follow-up	
	W-IPT	TAU	W-IPT	TAU	W-IPT	TAU
	Cohen's d	Cohen's d	Cohen's d	Cohen's d	Cohen's d	Cohen's d
Hamilton 24	1.246 [L]	0.255 [S]	1.115 [L]	0.381 [S]	0.007 [T]	0.096 [T]
Becks Depression Inventory	0.940 [L]	0.484 [S]	0.878 [L]	0.597 [M]	0.044 [T]	0.082 [T]
Work Ability Index	0.680 [M]	0.165 [S]	0.658 [M]	0.111 [T]	0.315 [S]	0.055 [T]
Return to Work	0.569 [M]	0.099 [S]	0.671 [M]	0.081 [T]	0.157 [T]	0.174 [T]
Insomnia Severity Index	1.145 [L]	0.212 [S]	1.177 [L]	0.382 [S]	0.179 [T]	0.150 [T]

T, trivial effect size; S, small effect size; M, medium effect size; L, large effect size.

### Symptoms of Depression: Experts’ Ratings

Experts' rated symptoms of depression decreased over time (large effect size), but more so in the W-IPT group compared to the TAU condition (medium effect size). Experts' rated symptoms of depression was lower in the W-IPT group (medium effect size). Within the W-IPT group, the symptoms of depression decreased from baseline to the end of the study (large effect size) and from baseline to follow-up (large effect size); from the end of the study to follow-up, the symptoms of depression remained stably low (trivial effect size). Within the TAU group, the symptoms of depression decreased from baseline to the end of the study (small effect size) and from baseline to follow-up (small effect size); from the end of the study to follow-up, the symptoms of depression remained stably low (trivial effect size).

### Symptoms of Depression: Self-Ratings

The self-rated symptoms of depression decreased in both groups over time (large effect size), but in the W-IPT group, the symptoms of depression decreased more compared to those of the TAU condition (small effect size). The self-rated symptoms of depression were lower in the W-IPT group (medium effect size). Within the W-IPT group, the symptoms of depression decreased from baseline to the end of the study (large effect size) and from baseline to follow-up (large effect size); from the end of the study to follow-up, the symptoms of depression remained stably low (trivial effect size). Within the TAU group, the symptoms of depression decreased slightly from baseline to the end of the study (small effect size) and from baseline to follow-up (medium effect size); from the end of the study to follow-up, the symptoms of depression remained stably high (trivial effect size).

### Ability to Work: Self-Ratings

The self-rated ability to work increased in both groups over time (medium effect size), but in the W-IPT group, the participants reported a higher self-rated ability to work compared to the TAU condition (medium effect size). The self-rated ability to work was higher in the W-IPT group (large effect size). Within the W-IPT group, the self-rated ability to work increased from baseline to the end of the study (medium effect size) and from baseline to follow-up (medium effect size); from the end of the study to follow-up, the ability to work remained stably low (small effect size). Within the TAU group, the self-rated ability to work increased slightly from baseline to the end of the study (small effect size) and from baseline to follow-up (trivial effect size); from the end of the study to follow-up, the ability to work remained stably low (trivial effect size) (cf. **Figure 2**).

### Return to Work: Self-Efficacy, Self-Ratings

The self-rated return to work increased in both groups over time (medium effect size), but in the W-IPT group, the self-rated return to work self-efficacy increased more compared to the TAU condition (medium effect size). The self-rated return to work was higher in the W-IPT group (medium effect size). Within the W-IPT group, the self-rated return to work increased from baseline to the end of the study (medium effect size) and from baseline to follow-up (medium effect size); from the end of the study to follow-up, return to work remained stably high (trivial effect size). Within the TAU group, the self-rated return to work increased slightly from baseline to the end of the study (small effect size) and from baseline to follow-up (trivial effect size); from the end of the study to follow-up, return to work remained stably low (trivial effect size).

### Subjective Sleep Quality

#### Insomnia Severity Index: Self-Ratings

The self-rated sleep complaints decreased in both groups over time (large effect size), but more so in the W-IPT group compared to the TAU condition (medium effect size). The self-rated sleep complaints were lower in the W-IPT group (medium effect size). Within the W-IPT group, the self-rated sleep complaints decreased from baseline to the end of the study (large effect size) and from baseline to follow-up (large effect size); from the end of the study to follow-up, the self-rated sleep complaints remained stably low (trivial effect size). Within the TAU group, the self-rated sleep complaints decreased only slightly from baseline to the end of the study (small effect size) and from baseline to follow-up (small effect size); from the end of the study to follow-up, the self-rated sleep complaints remained stably high (trivial effect size)

To summarize, the symptoms of depression (experts' rating) decreased in both groups over time (large effect sizes), but in the W-IPT group, the symptoms of depression decreased more compared to those of the TAU group. The reduction at follow-up led the W-IPT group to an altered assessment of the severity of the depression diagnosis (from moderate to mild), whereas in the TAU condition the reduction did not lead to a change in the assessment. The symptoms of depression (self-rating) decreased in both groups over time (large effect sizes), but in the W-IPT, the symptoms of depression decreased more compared to those of the TAU group. The work ability index self-rated interpretation gained in both groups over time (medium effect size), but in the W-IPT, the work ability increased more compared to that of the TAU group. The self-efficacy for the return to work gained in both groups over time (medium effect size), but in the W-IPT, the self-efficacy increased more compared to that of the TAU group. The self-rated symptoms of subjective sleep complaints decreased in both groups over time (large effect size), but in the W-IPT group, the sleep complaints decreased more compared to those of the TAU.

## Discussion

The key findings of the present study suggest that a specific work-related approach for depressed patients with work stress led to a greater decrease of clinician- and self-rated depressive symptoms compared to TAU. In addition, not only the subjective work ability was higher and the self-efficacy was more improved in the W-IPT group but also the quality of sleep was more increased compared to the TAU condition. Importantly, those effects remained stable over the follow-up time period. The particularly large effect in the W-IPT condition indicates that a job-related focus can help in the short and long terms.

The present study adds to the current literature in an important way because not only is the IPT beneficial for individuals with MDD (31, 55) but also it is especially based on psychosocial and workplace-related issues [see also Schramm and Berger (32)]. Our main study strength is the value of longitudinal research for extending knowledge about the relationship between W-IPT and MDD because of workplace-related stress.

The results are important for clinicians because the newest meta-analysis (8) showed that it is unclear whether the antidepressive treatment is more efficacious than the placebo treatment. There is an obvious necessity to use best evidence-based practices. The psychotherapeutic treatments are well evaluated, especially the cognitive–behavioral aspect, and IPT seems to be the preferred choice. The present study results are in line with the results shown before.

To summarize, all of our hypotheses plus the exploratory research question were fully confirmed. In this respect, comparing the effect of the intervention with those of the previous studies (see “Introduction”), again we hold that the present finding is clinically relevant. Thus, the present finding is in accord with previous results (33–35). We believe that the beneficial influence of W-IPT cannot be explained merely as an effect of time as they outperformed the outcome of the TAU condition.

In terms of comparing the long-term effect sizes of the present study with those of the previous studies, the present finding is in accord with previous results (37, 38).

We expected the work ability in the intervention group to be higher than in the TAU group. Surprisingly, the work ability in the W-IPT condition grew further even after the end of the study and over the follow-up time from originally poor to medium at the end of the intervention and almost good at follow-up, while the TAU condition showed a stable trend of poor work ability.

Despite the intriguing findings, several limitations warrant against overgeneralization of the present results. First, the sample size was small, although we basically relied on effect size calculations which, unlike *p*-values, do not vary with sample size, but since this was a pilot study, the small numbers can be explained. Further, there are increasing concerns about the importance and ‘significance' of *p*-values (56). Second, the sample with a broad age range can be seen not only as a possible limitation but also as a strong point. After all, there was a positive influence despite the heterogeneity of the sample.

Further studies might differentiate between specific groups (f.i., gender, age, or comorbidities). Another limitation is the relative short follow-up period of 3 months. A longer follow-up could be helpful, especially in practical uses (employee training). Booster sessions could be provided to ensure sustainable effects. In addition, our study compared the possible effect of a new group program with a TAU condition. We cannot definitively say whether or not it is superior to other evidence-supported psychological treatments such as cognitive behavioral therapy. Furthermore, the inclusion and exclusion criteria were chosen to identify a particular kind of sample. However, comorbidities such as substance use or personality disorders are common in depressive disorders. Lastly, it is possible that further latent but unassessed variables (family members, sleep–quality interaction, and increase in social support) might have biased the results shown (in any given direction possible).

## Conclusions

An 8-week group intervention with W-IPT had improved symptoms of depression, ability to work, self-efficacy regarding return to work, and sleep complaints more than the TAU condition.

We believe that the present results might be of broader interest: First, in terms of the costs for the companies (hiring new employee and absent or less productive employees) and for the employees (health and changing the job). Specifically, costs in employees being less productive or absent will result in several high costs per employee (26, 27, 30, 57). Second, the public health cost should also be mentioned here although we did not calculate the costs. Third, it follows that the benefit of an early investment in individual health should be the main interest of all three partners.

A short efficient treatment in groups seems to be easy to implement and economical. It could be a fixed part of a regular occupational health management (58, 59). The possible uses are (1) companies interested in having a modern occupational health management and (2) individuals experiencing stress and problems at the workplace for secondary or tertiary prevention.

## Data Availability Statement

The raw data supporting the conclusions of this article will be made available by the authors, without undue reservations, to any qualified researchers.

## Ethics Statement

The studies involving human participants were reviewed and approved by Ethikkommission Nordwest- und Zentralschweiz (EKNZ)—application number 2017-01-489. The patients/participants provided their written informed consent to participate in this study.

## Author Contributions

DN, NK, AK, SB, CW, NS, and UL wrote the proposal and designed the study and completed the final draft. DN, NK, AK, SB, CW, and NS were involved in data gathering and data entering. DN, NK, and SB performed the statistical analysis and wrote the draft. CW, NS, and UL commented on the second draft. All authors commented on the final manuscript, which was completed by DN, NK, AK, SB, CW, NS, and UL.

## Funding

This study was supported by the Thomi Hopf Stiftung, Oberwilerstrasse 65, 4123 Allschwil, Switzerland (www.thomi-hopf-stiftung.ch).

## Conflict of Interest

The authors declare that the research was conducted in the absence of any commercial or financial relationships that could be construed as a potential conflict of interest.
